# Bayesian mendelian randomization with study heterogeneity and data partitioning for large studies

**DOI:** 10.1186/s12874-022-01619-4

**Published:** 2022-06-03

**Authors:** Linyi Zou, Hui Guo, Carlo Berzuini

**Affiliations:** Centre for Biostatistics, School of Health Sciences, The University of Manchester, Oxford Road, Manchester, M13 9PL UK

**Keywords:** Mendelian randomization, Bayesian inference, Study heterogeneity, Data partitioning

## Abstract

**Background:**

Mendelian randomization (MR) is a useful approach to causal inference from observational studies when randomised controlled trials are not feasible. However, study heterogeneity of two association studies required in MR is often overlooked. When dealing with large studies, recently developed Bayesian MR can be computationally challenging, and sometimes even prohibitive.

**Methods:**

We addressed study heterogeneity by proposing a random effect Bayesian MR model with multiple exposures and outcomes. For large studies, we adopted a subset posterior aggregation method to overcome the problem of computational expensiveness of Markov chain Monte Carlo. In particular, we divided data into subsets and combined estimated causal effects obtained from the subsets. The performance of our method was evaluated by a number of simulations, in which exposure data was partly missing.

**Results:**

Random effect Bayesian MR outperformed conventional inverse-variance weighted estimation, whether the true causal effects were zero or non-zero. Data partitioning of large studies had little impact on variations of the estimated causal effects, whereas it notably affected unbiasedness of the estimates with weak instruments and high missing rate of data. For the cases being simulated in our study, the results have indicated that the “divide (data) and combine (estimated subset causal effects)” can help improve computational efficiency, for an acceptable cost in terms of bias in the causal effect estimates, as long as the size of the subsets is reasonably large.

**Conclusions:**

We further elaborated our Bayesian MR method to explicitly account for study heterogeneity. We also adopted a subset posterior aggregation method to ease computational burden, which is important especially when dealing with large studies. Despite the simplicity of the model we have used in the simulations, we hope the present work would effectively point to MR studies that allow modelling flexibility, especially in relation to the integration of heterogeneous studies and computational practicality.

## Background

Mendelian randomization (MR) [1–3] is a useful approach to causal inference from observational studies when randomised controlled trials are not feasible. It uses genetic variants as instrumental variables (IVs) to explore putative causal relationship between an exposure and an outcome. Conventional MR methods [4–11] have mainly used summary statistics of IV-exposure association and IV-outcome association analyses, from a single study (one-sample) or two independent studies (two-sample). Among recent developments of MR methods, a Bayesian approach [8, 12] has been proposed to tackle overlapping samples in which a subset of participants are common in the two association studies. This comes from the idea that overlapping- and two- sample settings can be treated as cases of missing data which can be imputed through Markov chain Monte Carlo (MCMC) while estimating causal effects of interest. This way, we take full advantage of all the observed and imputed data. Bayesian MR also offers great flexibility of modelling complex data structure and explicitly quantifies uncertainties of model parameters.

It is not uncommon that studies from different research groups are designed to address similar (but not exactly the same) scientific questions. For example, in a genome-wide association study (*Study* 1), data of genetic variants and hypertension status (outcome) are collected to identify outcome-associated genetic variants. In another independent study (*Study* 2), besides this aim, the investigator is also interested in causal effect of blood pressure medication (exposure) on hypertension. Therefore, exposure information is also recorded. To investigate the exposure-outcome causal relationship, a conventional option would be one-sample MR using data from *Study* 2 only. Another option would be a two-sample MR which will use genetic variants and the outcome data from *Study* 1, and genetic variants and the exposure data from *Study* 2. In other words, the outcome data of *Study* 2 will be discarded. Both of the options involve removal of data which, in our view, is not necessary. In fact, we can combine observed data from the two studies, and impute exposure data for *Study* 1 in a Bayesian MR model. However, it is well possible that the two studies are not homogenous, which should be taken into consideration in our modelling.

Another important aspect of Bayesian MR analysis (in fact, all kinds of data analysis) is tractability of computation, as we are in the era of big data. MCMC requires a large number of iterations and a complete scan of data for each iteration [13]. Thus, it can be computationally challenging, and sometimes even prohibitive. An intuitive solution would be dividing data into a number of subsets and enabling data analysis in parallel.

This paper aims to address study heterogeneity and data partitioning for large studies in Bayesian MR. First, we build a Bayesian MR model including multiple IVs, exposures and outcomes based on two independent studies, of which one has exposure data completely missing. To account for study heterogeneity, we propose a random effect model. Second, a data partitioning and subset posterior aggregation method [13] is adopted for analysis of large studies. Third, simulation experiments are carried out for different configurations of IV strength and missing rate of exposure data, followed by evaluation of our proposed method.

## Methods

### Bayesian MR with study heterogeneity

**Fig. 1:**
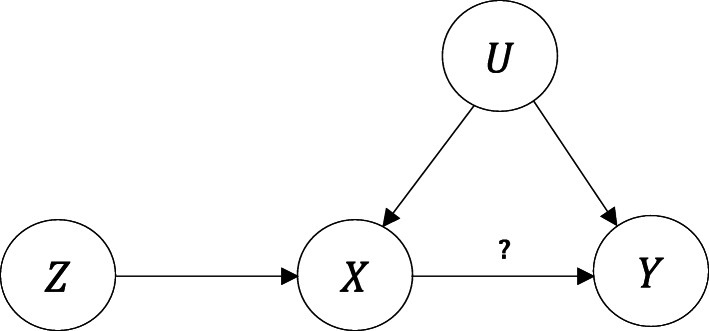
Schematic representation of the three assumptions required in Mendelian randomization

**Fig. 2:**
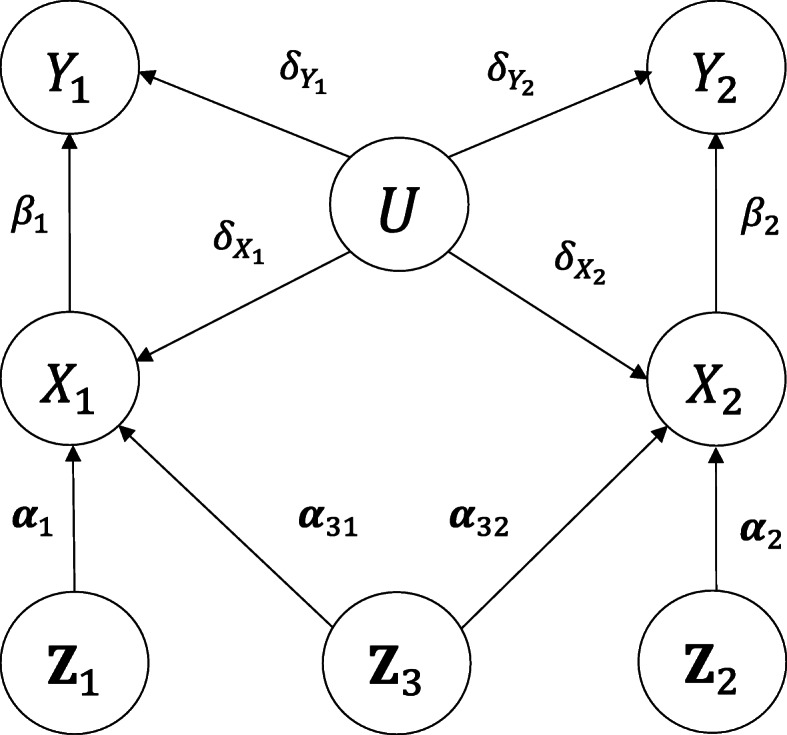
Graphical model of Mendelian randomisation with outcomes *Y*_1_ and *Y*_2_, exposures *X*_1_ and *X*_2_ and unobserved confounder *U*. **Z**_1_ consists of *L* instrumental variables of *X*_1_ and **Z**_2_ consists of *K* instrumental variables of *X*_2_. In addition, **Z**_3_ consists of *M* instrumental variables shared between *X*_1_ and *X*_2_. The instrumental variables are assumed to be mutually independent

Let *X* denote the exposure, *Y* the outcome, and *U* a scalar variable summarising the set of unobserved confounders of the relationship between *X* and *Y*. Traditional MR [9] requires that an IV (denoted by *Z*) is : *i*) associated with the exposure *X*, *ii*) not associated with the confounders *U*, and *iii*) associated with the outcome *Y* only through the exposure *X*. These three assumptions can be graphically expressed as Fig. 1 in which our interest is whether *X* causes *Y* (the *X*→*Y* arrow). For the purpose of illustration, we consider the data generating process shown in Fig. 2. **Z**_1_,**Z**_2_ and **Z**_3_ are vectors consisting of *L*,*K* and *M* independent IVs respectively. Random scalar variables *X*_1_ and *X*_2_ represent two exposures. Random scalar variables *Y*_1_ and *Y*_2_ represent two outcomes.

In a two-sample (or equivalently, two-study) MR setting with or without overlapping individuals, it has been shown that, compare to conventional MR analysis, a Bayesian approach may lead to more precise estimates of the causal effect by treating it as a case of incomplete data which may be dealt with through iterative imputations using MCMC [12]. Here, we further generalize the approach by allowing for some degree of heterogeneity between different studies.

Suppose we have data collected from two independent studies:

- *Study* *A* - observed data for IVs, exposures and outcomes {**Z**_1_,**Z**_2_,**Z**_3_,*X*_1_,*X*_2_,*Y*_1_,*Y*_2_}.
- *Study* *B* - observed data for IVs and outcomes {**Z**_1_,**Z**_2_,**Z**_3_,*Y*_1_,*Y*_2_} only.

*Study* *A* includes fully observed data for MR, whereas *Study* *B* has exposure data completely missing. We shall include random effect terms in our MR model to capture study heterogeneity. By assuming standardised observed variables and linear additivity, according to Fig. 2, our models are constructed as follows.

For *Study* *A*,

1 $$\begin{array}{*{20}l} U &\sim& N(0, 1), \end{array} $$

2 $$\begin{array}{*{20}l}  X_{1} | \mathbf{Z}_{1},\mathbf{Z}_{3},U &\sim& N\left(\boldsymbol{\alpha}_{1}\mathbf{Z}_{1} + \boldsymbol{\alpha}_{31}\mathbf{Z}_{3} + \delta_{X_{1}}U, \sigma_{X_{1A}}^{2}\right), \end{array} $$

3 $$\begin{array}{*{20}l}  X_{2} | \mathbf{Z}_{2},\mathbf{Z}_{3},U &\sim& N\left(\boldsymbol{\alpha}_{2}\mathbf{Z}_{2} + \boldsymbol{\alpha}_{32}\mathbf{Z}_{3} + \delta_{X_{2}}U, \sigma_{X_{2A}}^{2}\right), \end{array} $$

4 $$\begin{array}{*{20}l}  Y_{1} | X_{1},U &\sim& N\left(\beta_{1} X_{1}+\delta_{Y_{1}}U, \sigma_{Y_{1A}}^{2}\right), \end{array} $$

5 $$\begin{array}{*{20}l}  Y_{2} | X_{2},U &\sim& N\left(\beta_{2} X_{2}+\delta_{Y_{2}}U, \sigma_{Y_{2A}}^{2}\right). \end{array} $$

For *Study* *B*,

6 $$\begin{array}{*{20}l} U\! &\sim& \!N(0, 1), \end{array} $$

7 $$\begin{array}{*{20}l}  X_{1} | \mathbf{Z}_{1},\mathbf{Z}_{3},U\! &\sim& \!N\!\left(V_{X_{1}} \,+\, \boldsymbol{\alpha}_{1}\mathbf{Z}_{1} \,+\, \boldsymbol{\alpha}_{31}\mathbf{Z}_{3} \,+\, \delta_{X_{1}}U, \sigma_{X_{1B}}^{2}\right), \end{array} $$

8 $$\begin{array}{*{20}l}  X_{2} | \mathbf{Z}_{2},\mathbf{Z}_{3},U\! &\sim& \!N\!\left(V_{X_{2}} \,+\, \boldsymbol{\alpha}_{2}\mathbf{Z}_{2} \,+\, \boldsymbol{\alpha}_{32}\mathbf{Z}_{3} \,+\, \delta_{X_{2}}U, \sigma_{X_{2B}}^{2}\right), \end{array} $$

9 $$\begin{array}{*{20}l}  Y_{1} | X_{1},U\! &\sim& \!N\left(V_{Y_{1}} + \beta_{1} X_{1}+\delta_{Y_{1}}U, \sigma_{Y_{1B}}^{2}\right), \end{array} $$

10 $$\begin{array}{*{20}l}  Y_{2} | X_{2},U\! &\sim& \!N\left(V_{Y_{2}} + \beta_{2} X_{2}+\delta_{Y_{2}}U, \sigma_{Y_{2B}}^{2}\right). \end{array} $$

In the above pre-specified models, ***α***s are instrument strength parameters, and *δ*s are effects of *U* on *X*s or *Y*s. Causal effects of *X*s on *Y*s are denoted by *β*s. The study heterogeneity is accounted for by *V*s. Note that *X*_1_ and *X*_2_ do not have observed data in *Study* *B*, but they are part of data generating process, and thus, should be included in the model. *U* is a sufficient scalar summary of the unobserved confounders. We assume that *U*∼*N*(0,1).

The combined dataset of *StudiesA* and *B* ($\mathcal {D}$, say) will contain fully observed data for the instruments and the outcomes. However, all participants in *Study* *B* have missing data of *X*_1_ and *X*_2_ which will be treated as unknown quantities and imputed from their conditional distributions given the observed data and current estimated parameters using MCMC. Let *X*^∗^ be imputed values of *X*. Our approach involves the following sequence of five steps.

1. Specify initial values for unknown parameters and the number of Markov iterations *T*.
2. At the *t*th iteration, where 0≤*t*<*T*, let missing values of *X*_1_ and *X*_2_ in *Study* *B* be filled with $X_{1}^{*}$ drawn from $N\left (V_{X_{1}}^{(t)} + \boldsymbol {\alpha }_{1}^{(t)}\mathbf {Z}_{1} + \boldsymbol {\alpha }_{31}^{(t)}\mathbf {Z}_{3} + \delta _{X_{1}}^{(t)}U, {\sigma _{X_{1B}}^{2}}^{(t)}\right)$ and $X_{2}^{*}$ drawn from $N\left (V_{X_{2}}^{(t)} + \boldsymbol {\alpha }_{2}^{(t)}\mathbf {Z}_{2} + \boldsymbol {\alpha }_{32}^{(t)}\mathbf {Z}_{3} + \delta _{X_{2}}^{(t)}U, {\sigma _{X_{2B}}^{2}}^{(t)}\right)$, respectively. **Z**_1_,**Z**_2_ and **Z**_3_ are observed values of IVs in *Study* *B*.
3. Create a single complete dataset including both the observed and the imputed data.
4. Estimate model parameters using MCMC based on the complete dataset and set *t*←*t*+1.
5. Repeat Steps 2-4 until *t*=*T*.

Now we specify priors in the Bayesian model (2)-(10). Previous GWAS studies show that individual SNPs explain a tiny proportion of exposure variance [14–16], corresponding to small magnitudes of the ***α*** parameters in our model. In accord with this finding and previous MR simulation studies [17], we set IV strength parameters ***α***s to be independent and identically distributed with mean zero and a small variance: ***α***_1_∼*N*_*L*_(**0**,0.3^2^**I**),***α***_2_∼*N*_*K*_(**0**,0.3^2^**I**),***α***_31_∼*N*_*M*_(**0**,0.3^2^**I**), and ***α***_32_∼*N*_*M*_(**0**,0.3^2^**I**). The priors of both *β*_1_ and *β*_2_ are set to a same distribution *N*(0,10^2^). Finally, we assign the priors of the standard deviations *σ*s to a same inverse-gamma distribution *Inv-Gamma*(3,2), and random effects *V*s to *N*(0,1) in the Model (7)-(10) for *Study* *B*.

### Bayesian MR for large studies

Advantages of an MCMC-powered Bayesian approach to MR are counterpoised by a relatively higher computational burden and a possibly large memory requirement. A natural way of dealing with this problem would be to divide data $\mathcal {D}$ into a number (*J*, say) of subsets *D*_1_,*D*_2_,...,*D*_*J*_ that we assume to contain an equal number (*q*, say) of individuals for simplicity. By running separate Bayesian MR analyses in parallel on the subsets, we will obtain *J* subset-specific posteriors which can then be aggregated in various ways. In this study, we adopt a “divide-and-combine” approach proposed by Xue and Liang [13].

Let ***θ*** denote the entire set of unknown quantities in the model. For subset *D*_*j*_, where *j*=1,2,...,*J*, let *π*(***θ***|*D*_*j*_) denote the joint posterior distribution of ***θ*** and $\widehat {\boldsymbol {\mu }}_{j} = \widehat {E}(\boldsymbol {\theta }|D_{j})$ the corresponding estimated mean vector. Let $\widehat {\boldsymbol {\mu }} = \frac {1}{J}\sum _{j=1}^{J}\widehat {\boldsymbol {\mu }}_{j}$ be the average of the $\widehat {\boldsymbol {\mu }}_{j}$s. According to [13], the posterior based on full data, $\pi (\boldsymbol {\theta }|\mathcal {D})$, can be estimated as the average of the recentred subset posteriors.

11 $$\begin{array}{@{}rcl@{}} \widetilde{\pi}(\boldsymbol{\theta}|\mathcal{D}) = \frac{1}{J}\sum\limits_{j=1}^{J}\widetilde{\pi}(\boldsymbol{\theta} - \widehat{\boldsymbol{\mu}} + \widehat{\boldsymbol{\mu}}_{j} | D_{j}). \end{array} $$

And it has been proved that ([13])

12 $$ E_{\widetilde{\pi}}(\boldsymbol{\theta}) - E_{\pi}(\boldsymbol{\theta}) = O_{p}\left(q^{-1}\right),  $$

and

13 $$ Var_{\widetilde{\pi}}(\boldsymbol{\theta}) - Var_{\pi}(\boldsymbol{\theta}) = o_{p}\left(n^{-1}\right),  $$

where *q* is the sample size of the subsets and *n* the sample size of the full dataset. $E_{\widetilde {\pi }}(\boldsymbol {\theta })$ and *E*_*π*_(***θ***) are expectations of the posteriors of ***θ*** aggregated from subsets and obtained from full data respectively. $Var_{\widetilde {\pi }}(\boldsymbol {\theta })$ and *Var*_*π*_(***θ***) are their variances. It is easily seen that the difference in expectation depends on the sample size of the subsets and the difference in variation depends on the sample size of the full dataset.

### Simulations - Bayesian MR with study heterogeneity

We used simulated data to evaluate our Bayesian MR model with study heterogeneity in comparison with a conventional MR method. In particular, we considered 12 configurations including

- 3 missing rates of the exposures: 20%, 50%, 80%
- 2 degrees of the IV strength (***α***_1_,***α***_2_,***α***_31_,***α***_32_): **0.1** and **0.3**
- Zero and non-zero causal effects of the exposures on the outcomes (*β*_1_,*β*_2_): 0 and 0.3.

The number of IVs was set to 15, 15 and 5 for *Z*_1_,*Z*_2_ and *Z*_3_ respectively. Data of each IV were randomly drawn from a binomial distribution *B*(2,0.3) independently. The specified values of the effects of *U* on the exposures ($\delta _{X_{1}}, \delta _{X_{2}}$) and on the outcomes ($\delta _{Y_{1}}, \delta _{Y_{2}}$) were set to 1. Standard deviations *σ*s were set to 0.1. We simulated 200 datasets for each configuration.

For each dataset, we

- simulated a dataset of sample size *n*_*A*_ which contains observations of the IVs, exposures and outcomes (dataset *A*, denoted by $\mathcal {D}_{A}$);
- simulated a dataset of sample size *n*_*B*_ which contains observations of the IVs, exposures and outcomes, then included data of the IVs and outcomes only as if the exposure data were missing (dataset *B*, denoted by $\mathcal {D}_{B}$).

Sample size of $\mathcal {D}$, the combined data of $\mathcal {D}_{A}$ and $\mathcal {D}_{B}$, was set to 400 in all configurations, i.e., *n*=*n*_*A*_+*n*_*B*_=400. The missing rate of the exposures was defined as $\frac {n_{B}}{n}\times 100\%$. For example, if missing rate was 50%, we simulated $\mathcal {D}_{A}$ of sample size 200 and $\mathcal {D}_{B}$ of sample size 200. To allow for different degrees of study heterogeneity in different datasets, random effects *V*s in study *B* were randomly drawn from a uniform distribution *U*(−0.5,0.5) independently. Imputations of missing data and estimations of model parameters were then performed simultaneously using MCMC in Stan [18, 19]. $\hat {R}$ was used to check convergence of the Markov chains [20].

Estimated causal effects obtained from our Bayesian MR and two-sample inverse-variance weighted (IVW) estimation [6] were compared using 4 metrics: mean, standard deviation (sd), coverage (proportion of the times that the 95% credible/confidence intervals contained the true value of the causal effect) and power (proportion of the times that the 95% credible/confidence intervals did not contain zero when the true causal effect was non-zero, only applicable when *β*_1_=*β*_2_=0.3 by defination). Higher power indicates lower chance of getting false negative results. In IVW estimation, we used observed IV and exposure data from $\mathcal {D}_{A}$ and observed IV and outcome data from $\mathcal {D}_{B}$.

### Simulations - Bayesian MR with study heterogeneity for large studies

We also assessed the performance of dividing a big dataset into subsets in our Bayesian MR with study heterogeneity in simulation experiments. The simulation scheme was the same as above. However, the sample size of $\mathcal {D}$ was set to a much larger value 50,000. For each configuration, a single dataset was simulated by combining $\mathcal {D}_{A}$ and $\mathcal {D}_{B}$. We randomly divided data into 5 subsets of equal sample size, separately, for $\mathcal {D}_{A}$ ($\mathcal {D}_{A_{1}},..., \mathcal {D}_{A_{5}}$) and for $\mathcal {D}_{B}$ ($\mathcal {D}_{B_{1}},..., \mathcal {D}_{B_{5}}$). Subset $\mathcal {D}_{i}$ was then constructed by combining $\mathcal {D}_{A_{i}}$ and $\mathcal {D}_{B_{i}}$, where *i*=1,...,5. This is to ensure that subset $\mathcal {D}_{i}$ had the same missing rate as that of the full data $\mathcal {D}$. Causal effects were estimated using $\mathcal {D}$, and using the 5 subsets in Bayesian MR. To explore the impact of different data partitioning strategies on estimated causal effects, we carried out the same analysis by also dividing data into 50 subsets of sample size 1,000.

## Results

$\hat {R}$ values of all the parameters in the models (2)-(5) and (7)-(10) were greater than 1 and less than 1.1 across the simulations.

### Simulation results - Bayesian MR with study heterogeneity

Table 1 displays simulation results when the true causal effects were non-zero (*β*_1_=*β*_2_=0.3). Each row of the table corresponds to a configuration of a specified missing rate and a degree of IV strength ***α***. Columns correspond to the estimated causal effects of *X*_1_ on *Y*_1_ ($\hat {\beta }_{1}$) and of *X*_2_ on *Y*_2_ ($\hat {\beta }_{2}$) from our Bayesian method and from the IVW method evaluated using the four metrics. Unsurprisingly, the estimated causal effect of *X*_1_ on *Y*_1_ was very similar to that of *X*_2_ on *Y*_2_ in each configuration from Bayesian MR, because their true values were set to be the same and the model had a symmetric structure as shown in Fig. 2. This was also observed in the results from the IVW method. However, Bayesian MR outperformed IVW uniformly across all the configurations, with less bias, higher precision, coverage and power. The impact of low missing rate was positive on coverage but negative on power in IVW. However, such impact was negligible in Bayesian MR. This was mainly due to much higher variations of the estimates, and consequently, much wider confidence intervals in IVW estimation. Weaker IVs had little influence on unbiasedness of the estimates and power, but resulted in slightly lower precision and coverage in Bayesian MR. However, there was a remarkable decrease in unbiasedness, precision and power in IVW as IV strength decreased.

**Table 1:** Causal effects estimated from 200 simulated datasets for each configuration from two MR methods (Bayesian, IVW) when *β*_1_=*β*_2_=0.3, using four metrics: mean, standard deviation (sd), coverage and power. The six configurations were generated from three missing rates of the exposures (80%, 50%, 20%) and two levels of IV strength (***α***=**0.3** and **0.1**). $\hat {\beta }_{1}$: estimated causal effect of *X*_1_ on $Y_{1}, \hat {\beta }_{2}$: estimated causal effect of *X*_2_ on *Y*_2_

Missing rate	***α***	$\widehat {\beta _{1}}$								$\widehat {\beta _{2}}$							
		Bayesian				IVW				Bayesian				IVW			
		mean	sd	coverage	power	mean	sd	coverage	power	mean	sd	coverage	power	mean	sd	coverage	power
80%	0.3	0.299	0.005	0.980	1	0.217	0.101	0.790	0.685	0.298	0.005	0.970	1	0.209	0.086	0.765	0.665
	0.1	0.298	0.015	0.975	1	0.081	0.141	0.695	0.065	0.299	0.015	0.985	1	0.071	0.146	0.690	0.045
50%	0.3	0.300	0.004	0.975	1	0.245	0.118	0.920	0.580	0.299	0.004	0.980	1	0.265	0.113	0.935	0.595
	0.1	0.302	0.013	0.960	1	0.169	0.277	0.925	0.115	0.302	0.013	0.955	1	0.122	0.268	0.900	0.075
20%	0.3	0.299	0.004	0.970	1	0.260	0.203	0.915	0.255	0.299	0.004	0.970	1	0.276	0.185	0.955	0.285
	0.1	0.303	0.012	0.955	1	0.193	0.439	0.945	0.050	0.302	0.012	0.950	1	0.181	0.469	0.945	0.070

Table 2 presents simulation results when the true causal effects were zero (*β*_1_=*β*_2_=0). Again, the results of $\hat {\beta }_{1}$ was very similar to those of $\hat {\beta }_{2}$ in each configuration, separately, from Bayesian MR and from IVW. Overall, both methods performed well. However, Bayesian MR still outperformed IVW across all the configurations, with higher coverage and precision and less biased estimates. In both MR methods, missing rate did not have a notable effect on the estimates, whereas weaker IVs led to lower precision.

**Table 2:** Causal effects estimated from 200 simulated datasets for each configuration from two MR methods (Bayesian, IVW) when *β*_1_=*β*_2_=0, using four metrics: mean, standard deviation (sd), coverage and power. The six configurations were generated from three missing rates of the exposures (80%, 50%, 20%) and two levels of IV strength (***α***=**0.3** and **0.1**). $\hat {\beta }_{1}$: estimated causal effect of *X*_1_ on $Y_{1}, \hat {\beta }_{2}$: estimated causal effect of *X*_2_ on *Y*_2_

Missing rate	***α***	$\widehat {\beta _{1}}$						$\widehat {\beta _{2}}$					
		Bayesian			IVW			Bayesian			IVW		
		mean	sd	coverage	mean	sd	coverage	mean	sd	coverage	mean	sd	coverage
80%	0.3	-0.001	0.005	0.960	0.007	0.061	0.955	-0.001	0.005	0.955	-0.005	0.062	0.960
	0.1	0.004	0.016	0.960	-0.010	0.112	0.965	0.004	0.015	0.960	-0.001	0.130	0.960
50%	0.3	0.000	0.005	0.975	-0.014	0.087	0.935	0.000	0.005	0.955	-0.002	0.090	0.955
	0.1	0.004	0.013	0.970	0.005	0.188	0.960	0.004	0.013	0.955	-0.011	0.202	0.950
20%	0.3	0.000	0.004	0.950	0.010	0.148	0.930	0.000	0.004	0.965	-0.003	0.152	0.935
	0.1	0.003	0.012	0.965	0.012	0.394	0.920	0.003	0.012	0.965	0.020	0.361	0.945

### Simulation results - Bayesian MR with study heterogeneity for large studies

**Fig. 3:**
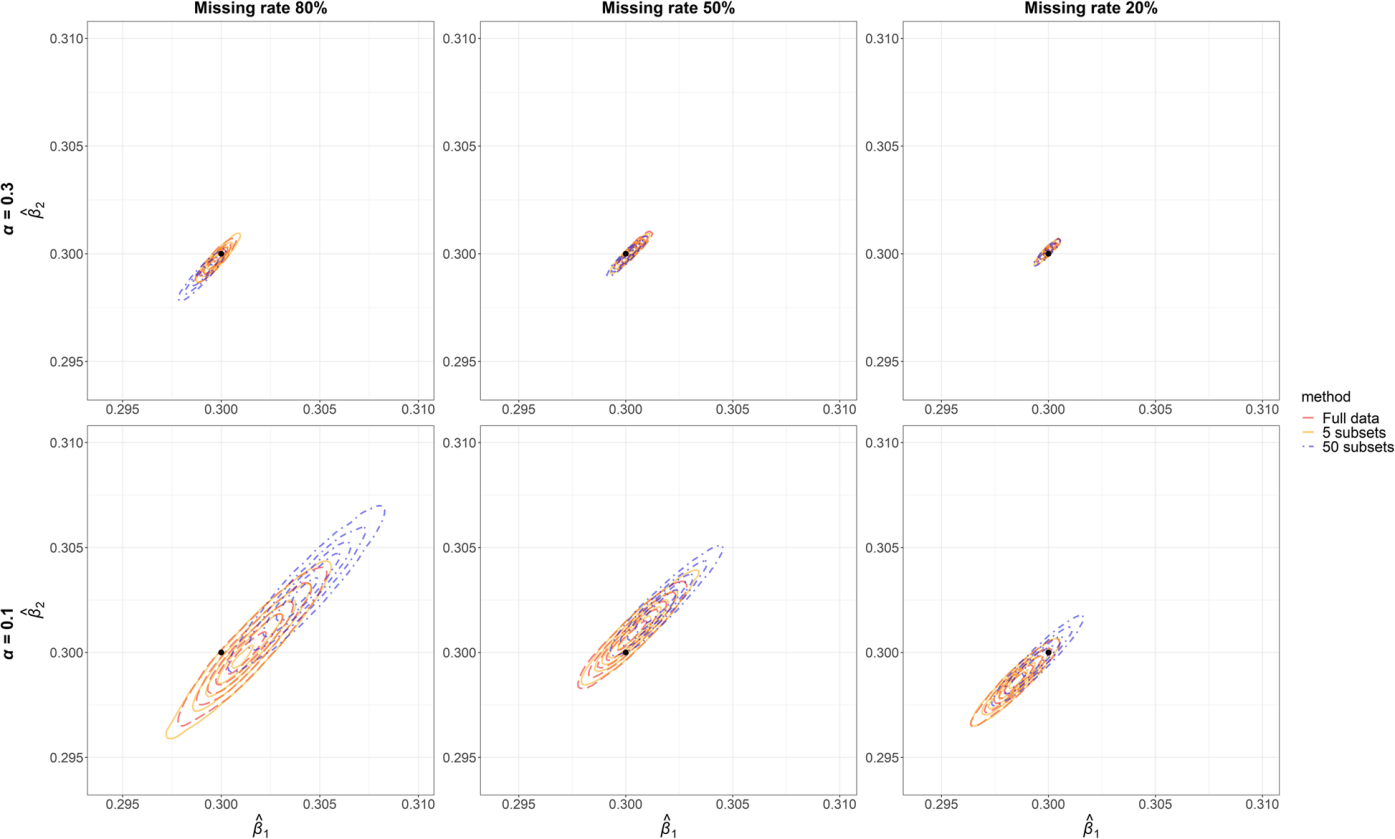
Joint posterior distributions of causal effects of *X*_1_ on *Y*_1_ ($\hat {\beta }_{1}$, horizontal axis) and *X*_2_ on *Y*_2_ ($\hat {\beta }_{2}$, vertical axis) obtained from 2-dimensional Gaussian kernel density estimation in Bayesian Mendelian randomisation when the true causal effects *β*_1_=*β*_2_=0.3. Results were based on full data (red), 5 subsets with equal sample size (orange) and 50 subsets with equal sample size (blue). ***α***: instrument strength

Figure 3 depicts the joint posterior distributions of $\hat {\beta }_{1}$ (horizontal axis) and $\hat {\beta }_{2}$ (vertical axis) based on simulated data when the true causal effects were non-zero. Columns corresponds to three missing rates and rows two levels of IV strength. In each panel, the black dot denotes the values of true causal effects (*β*_1_=*β*_2_=0.3). The red, orange and blue contours are 2-dimensional Gaussian kernel density estimation of the joint posterior (GKDEJP) from the full dataset, aggregated GKDEJP from five subsets and aggregated GKDEJP from fifty subsets respectively. When IVs were strong in Bayesian MR analysis (top panels), estimated causal effects were close to their true values, with or without data partitioning. When IVs became weaker (bottom panels), the results from the full data were concordant with those from 5 subsets, but notably different from those based on 50 subsets. The impact of data partitioning was substantial with weak IVs and high missing rate. This could be explained by Equation (12), in which difference in mean of the GKDEJPs depends on the subset sample size *q*. Difference in variance of the GKDEJPs was, however, not evident in the three sets of contours in each configuration, because it only depends on the sample size of the full data (Equation (13)) which was a fixed value 50,000. Our simulation results suggest that, in Bayesian MR with a large sample size, there is a trade-off between data partitioning for more efficient computations, and large enough sample size of each subset for preventing estimates from a decrease in unbiasedness.

**Fig. 4:**
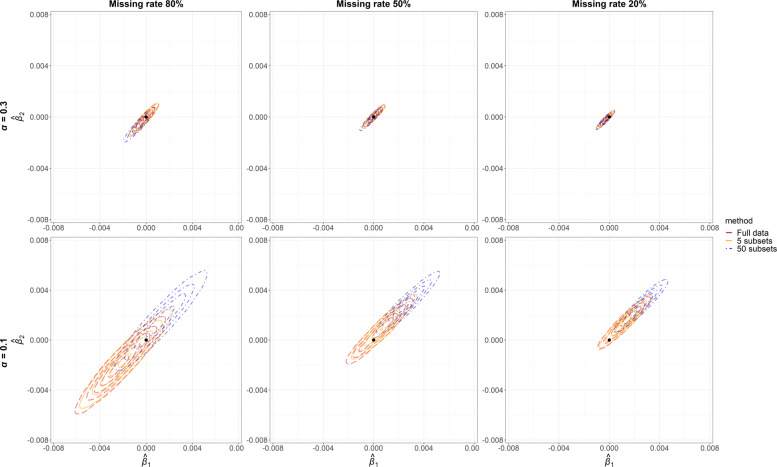
Joint posterior distributions of causal effects of *X*_1_ on *Y*_1_ ($\hat {\beta }_{1}$, horizontal axis) and *X*_2_ on *Y*_2_ ($\hat {\beta }_{2}$, vertical axis) obtained from 2-dimensional Gaussian kernel density estimation in Bayesian Mendelian randomisation when the true causal effects *β*_1_=*β*_2_=0. Results were based on full data (red), 5 subsets with equal sample size (orange) and 50 subsets with equal sample size (blue). ***α***: instrument strength

The same plots were presented in Fig. 4 when the true causal effects were zero. The performances of the three data partition strategies were very similar to those when the true causal effects were non-zero.

## Discussion and conclusions

Numerous MR methods have been developed in recent years. To the best of our knowledge, little attention has been focused on study heterogeneity. In this study, we further elaborated our Bayesian MR method [8, 12] by including random effects to explicitly account for study heterogeneity. We also adopted a subset posterior aggregation method [13] to address the computational challenge of MCMC, which is important especially when dealing with large studies. For the cases being simulated in our study, the results have indicated that the “divide (data) and combine (estimated subset causal effects)” can help improve computational efficiency, for an acceptable cost in terms of bias in the causal effect estimates, as long as the size of the subsets is reasonably large. However, when instruments are weak and data missing rate is high, the results obtained using data partitioning are noticeably different from those obtained using full data. Hence, there is room for further development of robust and computationally efficient methods for Bayesian MR.

Despite the simplicity of the model we have used in the simulations, we hope the present work would effectively point to MR studies that allow modelling flexibility, especially in relation to the integration of heterogeneous studies and computational practicality.

## Data Availability

The code of data simulations is available from the corresponding author upon request.

## Abbreviations

MR: Mendelian randomization
IV: Instrumental variable
MCMC: Markov chain Monte Carlo
IVW: Inverse-Variance Weighted
GKDEJP: Gaussian kernel density estimation of the joint posterior
